# Focused Training in Pelvic Floor Surgery: A CUSUM Analysis of the Learning Curve in Uterosacral Ligament Suspension

**DOI:** 10.3390/healthcare14152399

**Published:** 2026-08-05

**Authors:** Marta Barba, Alice Cola, Tomaso Melocchi, Desirèe De Vicari, Matteo Frigerio

**Affiliations:** Department of Gynecology, IRCCS San Gerardo dei Tintori, University of Milano-Bicocca, 20900 Monza, Italy; m.barba8792@gmail.com (M.B.);

**Keywords:** pelvic organs prolapse, native tissue repair, quality of life, elderly population, fragility, complications

## Abstract

Introduction: Pelvic organ prolapse (POP) involves the descent of pelvic organs into the vaginal cavity due to weakened pelvic support structures, particularly ligamentous and connective tissue laxity or defects. Surgical interventions are common, with approximately 200,000 procedures annually in the US. Surgical approaches prioritize apical support preservation, often using native tissue for reinforcement. The uterosacral ligament suspension (USLS) via vaginal approach offers advantages in cost and time. Presently, there is a resurgence of interest in transvaginal native-tissue procedures for pelvic floor reconstructive surgery, driven by factors such as cost-effectiveness and the desire to avoid complications associated with mesh. However, the declining use of this approach may result in insufficient exposure for residents and gynecologists to acquire vaginal surgical skills. Our study aims to comprehensively assess the learning curve associated with vaginal hysterectomy, considering the impact of experience on operative outcomes, complications, and patient results. Methods: This retrospective single-center study analyzes consecutive patients who underwent vaginal hysterectomy followed by high uterosacral ligament suspension for pelvic organ prolapse (POP) performed by one pelvic floor surgeon in training at Fondazione IRCCS San Gerardo dei Tintori Hospital in Monza, Italy, between November 2021 and February 2023. Patients underwent transvaginal hysterectomy and salpingectomy, with additional procedures as necessary, followed by high uterosacral ligament suspension. Follow-up assessments were performed periodically to evaluate symptoms and determine objective recurrence. Comparative analyses were conducted on preoperative POP-Q stages, demographic attributes, perioperative outcomes, and recurrence rates. Additionally, we utilized cumulative summation (CUSUM) analysis to examine the learning curve associated with vaginal hysterectomy accompanied by high uterosacral ligament suspension, focusing on surgical failure and operation duration. Results: A total of 43 patients were included in the study. The population had a median age of 62 years and a mean menopausal onset age of 49.7 years. Symptoms included bulging (100%), with 70% reporting bladder emptying difficulties. All patients exhibited stage II or higher anterior compartment prolapse, and the majority had stage II or higher central prolapse. Vaginal hysterectomy and uterosacral ligament suspension were performed on all patients, with additional repairs as needed. The median operative time was 119.45 ± 20.4 min, with a mean blood loss of 303.3 ± 107.9 mL. Postoperative assessment utilizing the POP-Q system revealed significant improvements in the anterior (*p* < 0.001 for Aa and Ba), central (*p* < 0.001 for C), and posterior (*p* < 0.001 for Ap and Bp) compartments compared to preoperative measures. Additionally, a decrease in the genital hiatus and an increase in the perineal body were noted (*p* < 0.001 for gh and pb). Functional outcomes demonstrated a substantial improvement in micturition symptoms and vaginal bulging compared to baseline (*p* < 0.001), although not all parameters reached statistical significance. Recurrence rates were 9.3% for the anterior compartment and 2.3% for the central compartment. The CUSUM analysis indicated proficiency in operation time after the 30th procedure, while surgical proficiency, defined by surgical success, entails no recurrence within 12 months postoperatively, was stabilized after 22 cases. Conclusions: Vaginal uterine ligament suspension not only stands as is a safe and effective procedure for pelvic organ prolapse but also boasts a brief learning curve, facilitating rapid enhancement in surgical proficiency within a condensed time frame, particularly in terms of blood loss, anatomical recurrence, and subsequent reintervention.

## 1. Introduction

Pelvic organ prolapse (POP) represents a common condition characterized by the descent of pelvic organs, such as the uterus, bladder, or rectum, into the vaginal canal as a consequence of weakened pelvic floor musculature and connective tissue. Reported prevalence varies widely, ranging from 3% to 50%, reflecting both methodological differences among studies and the underreporting of asymptomatic cases [1]. In the United States, approximately 200,000 surgical procedures are performed annually to address POP or associated urinary incontinence, with a relevant proportion requiring reintervention [2].

Accurate diagnosis relies on a thorough medical history, detailed physical examination, and, when indicated, adjunctive imaging techniques to define the type and severity of prolapse [3]. Although not invariably symptomatic, POP frequently manifests with pelvic pressure, dyspareunia, and functional disturbances of urinary and bowel function, with significant repercussions on quality of life, sexual function, and body image [4].

Management encompasses a spectrum of options, ranging from conservative measures—including pelvic floor muscle training, lifestyle modification, and pessary use—to surgical repair. Surgical intervention is generally reserved for symptomatic women who fail conservative treatment. Among surgical strategies, restoration of apical support constitutes a cornerstone of durable repair. Native tissue approaches have gained renewed interest due to their cost-effectiveness and avoidance of mesh-related complications. In this context, uterosacral ligament suspension (USLS) is recognized as a reliable procedure, ensuring dual apical reinforcement [5,6].

The vaginal route offers further advantages, being associated with reduced operative time, lower costs, and fewer complications compared to abdominal or laparoscopic approaches. Mean operative times are estimated at 46.6 min for the vaginal route, versus 55.2 min for the abdominal and 82.5 min for laparoscopic procedures [7,8]. Even within reconstructive techniques such as USLS, vaginal access demonstrates greater procedural efficiency. Indeed, laparoscopic USLS is associated with longer operative times, averaging 120.6 min, while vaginal hysterectomy (VH) consistently outperforms total laparoscopic hysterectomy (TLH), with a mean operative time difference of approximately 42 min [9,10].

Although vaginal surgery remains a valuable option for the treatment of pelvic organ prolapse, its utilization has progressively decreased with the widespread adoption of laparoscopic and robotic approaches. As a consequence, opportunities for trainees to gain hands-on experience in vaginal hysterectomy and apical suspension procedures have become increasingly limited, raising concerns about the preservation of essential vaginal surgical skills and the future availability of adequately trained surgeons.

Despite the well-established role of vaginal hysterectomy with uterosacral ligament suspension in the treatment of pelvic organ prolapse, evidence regarding the learning process required to achieve consistent surgical proficiency remains scarce. Most studies have focused on anatomical and functional outcomes, whereas the educational aspects of the procedure have received comparatively little attention. In the current era, characterized by reduced exposure to vaginal surgery and increasing adoption of laparoscopic and robotic techniques, understanding the learning curve of this procedure is essential to optimize training pathways and ensure maintenance of vaginal reconstructive surgical skills.

There is a renewed interest among scholars in transvaginal native-tissue procedures, particularly for pelvic floor reconstructive surgery, primarily driven by considerations of cost-effectiveness and the avoidance of complications associated with mesh application [11]. Nevertheless, the utilization of the transvaginal approach is declining, resulting in reduced exposure of residents to vaginal hysterectomies and, consequently, diminished confidence in performing vaginal surgery. Surgeons increasingly convert vaginal hysterectomies to laparoscopic or robotic procedures, further limiting trainee exposure. Moreover, there are no established data regarding the minimum number of procedures required to complete the learning curve for vaginal hysterectomy.

Accordingly, this study aimed to evaluate the learning curve for vaginal hysterectomy in a single-surgeon case series, analyzing the impact of surgical experience on operative metrics, complications, objective cure rates, and both subjective and functional outcomes.

## 2. Materials and Methods

This retrospective single-center study analyzed consecutive patients undergoing vaginal hysterectomy followed by high uterosacral ligament (USL) suspension for pelvic organ prolapse (POP), performed by a single pelvic floor surgeon in training (MB) at Fondazione IRCCS San Gerardo dei Tintori Hospital, Monza, Italy, between November 2021 and February 2023. The surgeon in training had prior limited experience restricted to obstetric tear repairs and episiotomy suturing, with no prior experience in vaginal hysterectomy or prolapse surgery.

Preoperative assessment included a medical interview to document obstetric history and evaluation of urinary, sexual, and bowel symptoms according to the standardized terminology of the International Urogynecology Association and the International Continence Society [12]. Urogenital examination and POP staging were performed using the Pelvic Organ Prolapse Quantification (POP-Q) system [13].

**Figure 1:**
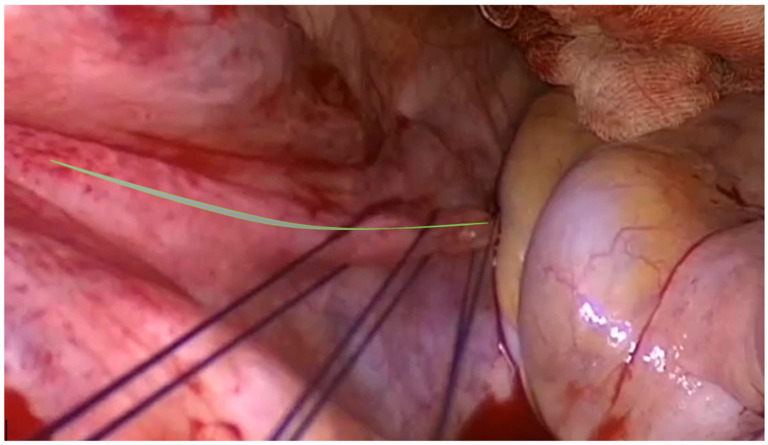
High uterosacral ligament transfixion (green line)–right side. Image was acquired for didactical purpose through a laparoscopic camera by vaginal route to better demonstrate the USL transfixion.

Patients underwent transvaginal hysterectomy with salpingectomy; bilateral oophorectomy was performed when feasible based on menopausal status, age, oncologic risk, and patient preference. All procedures were assisted by experienced pelvic floor surgeons. Apical suspension was performed via high USL suspension using two or three monofilament absorbable size 0 sutures per side as previously described (Figure 1) [14]. Additional anterior and/or posterior repairs were performed as indicated [15,16,17].

Follow-up occurred at 1 month, 6 months, 1 year, and annually thereafter, and included a clinical interview and a urogenital examination. Subjective recurrence was defined by postoperative bulging symptoms; objective recurrence was defined by POP-Q stage ≥ II or reoperation [18,19,20].

The study was approved by the Institutional Review Board of San Gerardo Hospital (SH-MCC 1709/2013). Data were collected using hospital software, entered by one author, and verified by another. Statistical analyses were conducted with statistical software JMP (Version 7; SAS Institute Inc., Cary, NC, USA), including descriptive statistics, trend visualization, analysis of variance (F test), and linear regression. *p* < 0.05 was considered significant. The cumulative sum (CUSUM) method was used to evaluate the learning curve by plotting the cumulative differences between individual values and the overall mean.

## 3. Results

During the study period, 47 patients underwent vaginal hysterectomy with high uterosacral ligament suspension for pelvic organ prolapse (POP). Four patients were excluded due to loss to follow-up, leaving 43 patients for analysis. Patient characteristics are summarized in Table 1. The median age was 62 years, with mean age at menopause of 49.7 years; only three patients were on hormone replacement therapy. More than half (22/43) were sexually active. Baseline symptoms and POP-Q measurements are detailed in Table 2. All patients had stage II or higher anterior compartment prolapse, most also had stage II or higher central prolapse, and all reported bulging symptoms. Approximately 70% experienced voiding difficulties, with stress urinary incontinence in 27.9%, overactive bladder in 25.6%, and urge incontinence in 16.3%.

All patients underwent vaginal hysterectomy with apical suspension, predominantly via uterosacral ligament suspension (95.3%); McCall culdoplasty was performed in 2 patients (4.6%). Anterior repair was performed in 97.6%, posterior repair in 13.95%. Median operative time was 119.45 ± 20.4 min with mean blood loss of 303.3 ± 107.9 mL and mean hemoglobin decrease of 1.47 ± 0.99 g/dL. Only three patients had ASA scores ≥ 3 (Table 3). Mean follow-up was 10.2 ± 8.3 months, during which two complications occurred (intestinal and urethral). Recurrence rates were 9.3% (anterior), 2.3% (central), and 0% (posterior); none required reoperation (Table 4).

POP-Q comparisons demonstrated significant improvements in the anterior (Aa, Ba; *p* < 0.001), central (C; *p* < 0.001), and posterior (Ap, Bp; *p* < 0.001) compartments, with decreased genital hiatus and increased perineal body (*p* < 0.001) (Table 5). Functional outcomes showed significant improvement in bulging and micturition symptoms (*p* < 0.001) (Table 6).

**Figure 2:**
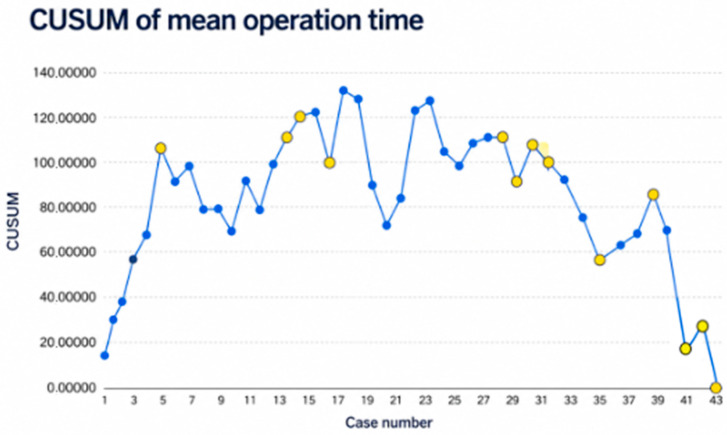
Control chart for variables, CUSUM chart, which reports the cumulative sum of differences from a specified value, aimed at determining competency in terms of surgical time. The *x*-axis indicates the procedure ordered in numerical sequence. The *y*-axis represents the CUSUM statistic, defined as the cumulative sum of deviations from the target operative time and therefore reflecting the progressive deviation from expected performance over time. Yellow dots denote procedures during which a bilateral adnexectomy was also performed.

**Figure 3:**
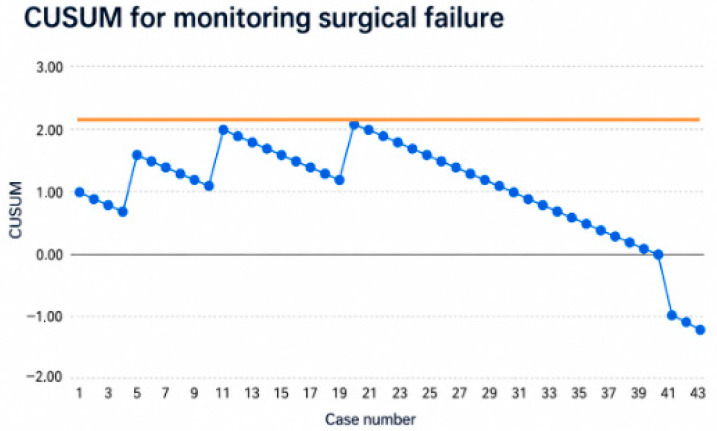
The cumulative sum (CUSUM) control chart analysis of surgical failures is depicted with the x-axis representing the number of procedures performed. The curve increases by the current mean probability of failure (11.1%) when a surgical failure occurs, and decreases by the complementary probability when a success occurs. This CUSUM control chart analysis is predicated on an acceptable failure rate of 10% and an unacceptable failure rate of 20%. The threshold for unacceptable recidivism, indicated by the orange line, is presented as a horizontal line at 2. A consistent success rate below this line indicates achievement of competency.

CUSUM analysis of operative time showed no improvement during the first five procedures, with noticeable enhancement after the 20th case and proficiency achieved by the 30th case (Figure 2). CUSUM analysis of surgical failure was performed using predefined performance thresholds, with an acceptable failure rate of 10% and an unacceptable rate of 20%. The CUSUM chart was constructed to monitor performance over sequential cases, with the x-axis representing the chronological order of procedures. The y-axis represents the CUSUM statistic, defined as the cumulative sum of risk-adjusted deviations between observed outcomes and expected performance. For each case, the CUSUM value was updated by adding a positive increment in the event of surgical failure and a negative increment in the event of success, with increments weighted according to the probability model derived from the predefined acceptable and unacceptable failure rates (mean estimated failure probability 11.1%). Thus, the y-axis reflects a continuous measure of deviation from expected performance rather than a direct proportion or probability of failure. A control limit (orange line at 2) was used to define unacceptable performance, and sustained values below this threshold were considered indicative of achievement of competency. Performance stabilization was observed after 22 cases, corresponding to consistent outcomes below the control limit and absence of recurrence within 12 months (Figure 3).

## 4. Discussion

This study demonstrates the efficacy and safety of vaginal hysterectomy with high uterosacral ligament suspension (USLS) for the management of pelvic organ prolapse (POP). Significant anatomical improvements were observed across all compartments, as indicated by enhanced POP-Q measurements in the anterior (Aa, Ba), apical (C), and posterior (Ap, Bp) sites (*p* < 0.001), along with reduction in the genital hiatus and increased perineal body length, reflecting restoration of pelvic floor support and function.

Recurrence rates—9.3% for the anterior compartment and 2.3% for the apical compartment—align with published outcomes for native tissue repairs, supporting the durability and clinical viability of vaginal USLS when performed by trained and experienced surgeons. This is particularly relevant given the declining use of mesh-based interventions due to safety concerns and evolving regulatory guidelines [18,21].

A key contribution of this study is the assessment of the surgical learning curve for vaginal hysterectomy and USLS. Cumulative summation (CUSUM) analysis revealed a plateau in operative time after the 30th procedure, with surgical proficiency—defined as absence of recurrence within 12 months—achieved after 22 cases, consistent with prior validation of CUSUM methodology by Wu et al. [2]. These findings suggest that efficient and reliable clinical outcomes can be achieved within a relatively brief, structured, and supervised training period.

However, it is important to emphasize that the definition of “surgical proficiency” used in this study—based primarily on recurrence within a defined follow-up period—represents only a partial measure of competence. A more comprehensive definition of proficiency should also include perioperative complications, intraoperative technical difficulty, need for senior assistance, and procedural autonomy, all of which may evolve independently of recurrence outcomes.

From an educational perspective, these findings underscore a growing concern: the progressive loss of vaginal surgical exposure in contemporary gynecologic training pathways. As minimally invasive abdominal and robotic techniques become dominant [8], opportunities to acquire and maintain vaginal surgical skills are diminishing. This raises the risk that future surgeons may lack proficiency in procedures such as vaginal hysterectomy and USLS, despite their continued clinical relevance, particularly in high-risk or resource-limited settings.

Although transvaginal USLS offers advantages in cost, learning curve, and short-term efficacy, it has limitations including dependence on ligament quality, limited ureteral visualization, and technical sensitivity to suspension placement. Intraoperative cystoscopy is recommended in trainees or complex cases to ensure ureteral safety. Alternative approaches such as vNOTES or robotic surgery may improve visualization and suturing accuracy, particularly in high-risk or recurrent patients.

One limitation of this study is its retrospective, single-surgeon design, which may restrict the generalizability of the findings. Nonetheless, the consistency of surgical technique and operator enhances the reliability of the learning curve analysis and procedural outcomes. Future research should include prospective, multicenter studies to validate these results and further explore long-term functional and patient-reported outcomes.

An additional consideration is the relatively short mean follow-up (~10 months), which may underestimate long-term recurrence rates, particularly for apical prolapse. While early outcomes are encouraging, longer follow-up is necessary to confirm durability of repair and stability of the learning curve beyond the early postoperative period. This limitation should be considered when interpreting recurrence and durability outcomes.

Case selection may also have influenced the observed learning curve. As is typical in surgical training, early cases were likely more standardized and less complex, whereas progressively more challenging cases may have been introduced as the surgeon gained confidence and autonomy. This natural evolution in case complexity may partially confound the apparent improvement in operative metrics and should be considered when interpreting the slope and plateau of the learning curve.

The acquisition of surgical competence is a multifactorial process that extends beyond procedural repetition alone. Technical skills, anatomical knowledge, intraoperative decision-making, and the ability to manage unexpected findings all contribute to the progressive development of surgical expertise. In pelvic floor reconstructive surgery, these aspects are particularly relevant because procedural success depends not only on technical execution but also on appropriate patient selection and a thorough understanding of pelvic support anatomy.

As opportunities for training in vaginal surgery have decreased over recent decades, concerns have emerged regarding the preservation of these skills among new generations of gynecologic surgeons. Consequently, there is increasing interest in identifying objective measures of surgical progression and defining realistic training targets. Quantitative assessment of performance can help training programs determine the number of procedures required to achieve competency, optimize supervision strategies, and ensure adequate exposure during residency and fellowship.

In urogynecology, several studies have explored the acquisition of proficiency for different diagnostic and therapeutic procedures, highlighting how surgical performance tends to improve progressively with accumulated experience. Evaluating these patterns may provide valuable information for curriculum development and for maintaining high standards of patient care while preserving expertise in vaginal reconstructive surgery.

## 5. Conclusions

Vaginal hysterectomy with high uterosacral ligament suspension is a safe and effective procedure for pelvic organ prolapse, associated with good anatomical and symptomatic outcomes and low complication rates. The learning curve is brief, with proficiency typically achieved after 22–30 cases. Structured training and supervised practice are essential to preserve these skills in future gynecologic surgeons, ensuring that vaginal USLS remains a cornerstone of pelvic reconstructive surgery.

## Figures and Tables

**Table 1:** **Patients’ characteristics**. Continuous data as mean ± standard deviation (SD); non-continuous data as absolute numbers with percentages.

Mean Age	62 ± 9.3
Parity	2 ± 0.7
Menopause	39 (90.6%)
Patients on HRT	3 (6.9%)
Mean age of menopause onset	49.7 ± 3.2
Sexual active	22 (51.1%)

**Table 2:** **Baseline data**: Continuous variables are presented as mean ± SD; categorical variables as n (%). POP-Q points: Aa, fixed mid-anterior wall 3 cm from urethral meatus; Ba, most distal anterior wall point; C, vaginal cuff edge (post-hysterectomy); gh, genital hiatus; pb, perineal body; tvl, total vaginal length; Ap, fixed mid-posterior wall 3 cm from hymen; Bp, most distal posterior wall point.

Stress Urinary incontinence	12 (27.9%)
Overactive bladder syndrome	11 (25.6%)
Urge urinary incontinence	7 (16.3%)
Bulging symptoms	43 (100%)
Voiding symptoms	29 (67.4%)
Constipation	10 (23.2%)
Aa	+1.59 ± 1.94
Ba	+1.63 ± 1.96
C	+1.69 ± 2.36
gh	4.20 ± 0.63
pb	2.58 ± 0.54
tvl	9.55 ± 0.85
Ap	−1.79 ± 0.91
Bp	−1.79 ± 0.91
D	−4.18 ± 1.31

**Table 3:** **Operative data**. Continuous data as mean ± SD; non-continuous data as absolute numbers with percentages.

Vaginal hysterectomy	43 (100%)
Uterosacral ligaments suspension	41 (95.3%)
McCall culdoplasty	2 (4.6%)
Anterior repair	42 (97.6%)
Posterior repair	6 (13.95%)
Mean Blood Loss	303.3 ± 107.9
ASA score ≥ 3	3 (6.9%)
Pre-op & Post-op Hb Δ	−1.47 ± 0.99
Mean operative time in minutes	119.45 ± 20.4
Surgical complication (urethral and intestinal)	2 (4.6%)

**Table 4:** **Objective outcomes**. Continuous data as mean ± SD; non-continuous data as absolute numbers with percentages.

Mean follow-up time (months)	10.2 ± 8.3
Anterior compartment relapse	4 (9.3%)
Central compartment relapse	1 (2.3%)
Posterior compartment relapse	0
Addition treatment (reoperation, pessary, sling)	0

**Table 5:** Anatomic outcomes (POP-Q): Aa, fixed point on mid-anterior vaginal wall 3 cm from external urethral meatus; Ba, most distal anterior wall point; C, vaginal cuff edge (post-hysterectomy); gh, genital hiatus; pb, perineal body; tvl, total vaginal length; Ap, fixed point on mid-posterior wall 3 cm from hymen; Bp, most distal posterior wall point. Data presented as mean ± SD.

	Preoperative	Postoperative	*p*-Value
Aa	+1.59 ± 1.94	−2.48 ± 0.66	<0.0001
Ba	+1.63 ± 1.96	−2.44 ± 0,74	<0.0001
C	+1.69 ± 2.36	−7.81 ± 1.51	<0.0001
gh	4.20 ± 0.63	3.51 ± 0.50	<0.0001
pb	2.58 ± 0.54	2.98 ± 0.23	<0.0001
tvl	9.55 ± 0.85	9.18 ± 0.98	0.0220
Ap	−1.79 ± 0.91	−2.53 ± 0.66	<0.0001
Bp	−1.79 ± 0.91	−2.53 ± 0.66	<0.0001
D	−4.18 ± 1.31	/	n/A

**Table 6:** Functional outcomes. Data as absolute numbers with percentages.

	Preoperative	Postoperative	*p*-Value
Stress urinary incontinence	12 (27.9%)	12 (27.9%)	0.5
Overactive bladder syndrome	11 (25.6%)	7 (16.3%)	0.1048
Urge urinary incontinence	7 (16.3%)	3 (6.9%)	0.0798
Voiding symptoms	29 (67.4%)	8 (12.3%)	<0.0001
Sexual active	22 (51.1%)	28 (65.1%)	0.0415
Constipation	10 (23.2%)	8 (18.6%)	0.1451
Bulging symptoms	43 (100%)	3 (6.9%)	<0.0001

## Data Availability

The underlying data are not publicly available due to privacy and ethical restrictions related to patient confidentiality. However, anonymized data supporting the findings of this study are available from the corresponding author upon reasonable request.

## References

[1] Brown H.W., Hegde A., Huebner M., Neels H., Barnes H.C., Marquini G.V., Mukhtarova N., Mbwele B., Tailor V., Kocjancic E. (2022). International urogynecology consultation chapter 1 committee 2: Epidemiology of pelvic organ prolapse: Prevalence, incidence, natural history, and service needs. Int. Urogynecol. J..

[2] Wu J.M., Hundley A.F., Fulton R.G.B., Myers E.R. (2009). Forecasting the Prevalence of Pelvic Floor Disorders in U.S. Women: 2010 to 2050. Obstet. Gynecol..

[3] Raju R., Linder B.J. (2021). Evaluation and Management of Pelvic Organ Prolapse. Mayo Clin. Proc..

[4] Committee on Practice Bulletins—Gynecology and the American Urogynecologic Society (2017). Practice Bulletin No. 176: Pelvic Organ Prolapse. Obstet. Gynecol..

[5] Yilmaz E.P.T., Yapca O.E., Topdagi Y.E., Al R.A., Kumtepe Y. (2021). Comparison of two natural tissue repair-based surgical techniques; sacrospinous fixation and uterosacral ligament suspension for pelvic organ prolapse treatment. J. Gynecol. Obstet. Hum. Reprod..

[6] DeLancey J.O.L. (2016). What’s new in the functional anatomy of pelvic organ prolapse?. Curr. Opin. Obstet. Gynecol..

[7] Garry R., Fountain J., Mason S., Hawe J., Napp V., Abbott J., Clayton R., Phillips G., Whittaker M., Lilford R. (2004). The eVALuate study: Two parallel randomised trials, one comparing laparoscopic with abdominal hysterectomy, the other comparing laparoscopic with vaginal hysterectomy. BMJ.

[8] Douligeris A., Kathopoulis N., Zachariou E., Mortaki A., Zacharakis D., Kypriotis K., Chatzipapas I., Protopapas A. (2024). Laparoscopic Versus Vaginal Uterosacral Ligament Suspension in Women With Pelvic Organ Prolapse: A Systematic Review and Meta-Analysis of the Literature. J. Minim. Invasive Gynecol..

[9] Ronsini C., Pasanisi F., Cianci S., Vastarella M.G., Pennacchio M., Torella M., Ercoli A., Colacurci N. (2023). Laparoscopic uterosacral ligament suspension: A systematic review and meta-analysis of safety and durability. Front. Surg..

[10] Sandberg E.M., Twijnstra A.R., Driessen S.R., Jansen F.W. (2017). Total Laparoscopic Hysterectomy Versus Vaginal Hysterectomy: A Systematic Review and Meta-Analysis. J. Minim. Invasive Gynecol..

[11] Aarts J.W.M., E Nieboer T., Johnson N., Tavender E., Garry R., Mol B.W.J., Kluivers K.B. (2015). Surgical approach to hysterectomy for benign gynaecological disease. Cochrane Database Syst. Rev..

[12] Haylen B.T., de Ridder D., Freeman R.M., Swift S.E., Berghmans B., Lee J., Monga A., Petri E., Rizk D.E., Sand P.K. (2010). An International Urogynecological Association (IUGA)/International Continence Society (ICS) joint report on the terminology for female pelvic floor dysfunction. Neurourol. Urodyn..

[13] Bump R.C., Mattiasson A., Bø K., Brubaker L.P., DeLancey J.O., Klarskov P., Shull B.L., Smith A.R. (1996). The standardization of terminology of female pelvic organ prolapse and pelvic floor dysfunction. Am. J. Obstet. Gynecol..

[14] Spelzini F., Frigerio M., Manodoro S., Interdonato M.L., Cesana M.C., Verri D., Fumagalli C., Sicuri M., Nicoli E., Polizzi S. (2017). Modified McCall culdoplasty versus Shull suspension in pelvic prolapse primary repair: A retrospective study. Int. Urogynecology J..

[15] Manodoro S., Frigerio M., Milani R., Spelzini F. (2018). Tips and tricks for uterosacral ligament suspension: How to avoid ureteral injury. Int. Urogynecol. J..

[16] Beer F., Schaufelberger P., Friedl T.W.P., Lindner A., Schütze S., Janni W., Deniz M. (2026). Outcomes and patient satisfaction after pelvic organ prolapse surgery with and without mesh: A retrospective cohort study with prospective follow-up. Arch. Gynecol. Obstet..

[17] Menefee S.A., Richter H.E., Myers D., Moalli P., Weidner A.C., Harvie H.S., Rahn D.D., Meriwether K.V., Paraiso M.F.R., Whitworth R. (2024). Apical Suspension Repair for Vaginal Vault Prolapse: A Randomized Clinical Trial. JAMA Surg..

[18] Srikrishna S., Robinson D., Cardozo L. (2010). Validation of the Patient Global Impression of Improvement (PGI-I) for urogenital prolapse. Int. Urogynecol. J..

[19] Spelzini F., Frigerio M., Regini C., Palmieri S., Manodoro S., Milani R. (2017). Learning curve for the single-incision suburethral sling procedure for female stress urinary incontinence. Int. J. Gynecol. Obstet..

[20] Park K., Kwon J.Y., Yoo E.-H., Lee S.H., Song J.M., Pyeon S.Y. (2024). Application of CUSUM analysis in assessing learning curves in robot-assisted sacrocolpopexy performed by experienced gynecologist. BMC Surg..

[21] Yeung E., Baessler K., Christmann-Schmid C., Haya N., Chen Z., A Wallace S., Mowat A., Maher C. (2024). Transvaginal mesh or grafts or native tissue repair for vaginal prolapse. Cochrane Database Syst. Rev..

